# Supplementary material for: Rush order containment of critical drugs in ICUs
Source: PLoS One. 2022 Jun 23;17(6):e0264928. doi: 10.1371/journal.pone.0264928 (PMC9223342; doi:10.1371/journal.pone.0264928)
Supplement: Attachment — Submitted filename: reviewReportRev_PLOSONE.pdf [file pone.0264928.s001.pdf]

Dear reviewers,

We would like to inform you that before reviewing this manuscript we contacted the Editor. In our letter to the Editor, we explained the reasons that prevent us from thoroughly addressing some of your concerns (see comments 1.8 and 2.3 in Table 1) and we asked whether we could submit a revised version of the manuscript where some of your critical comments were not dealt with.
The Editor contacted the Academic Editor, who, after reviewing our motivations, encouraged us to:

1) submit a revised version of the paper not addressing the comments we were not able to deal with,
2) copy paste the emails we exchanged in the rebuttal letter to inform you about the decision taken
3) provide detailed answers to your concerns.

Based on the encouraging words of the Academic Editor, we have thoroughly revised our manuscript (the paragraphs that have been added are written in blue, we added a few pages (Section 4 was totally rewritten) incorporating most of the changes you suggested and we have provided a detailed answer to each of your concerns (see Table 1).

Here is the message we sent to the Academic Editor:

Dear Editor,
first of all, we would like to thank you for the opportunity to review our work. We also thank the Reviewers for their helpful and valuable comments. We believe that we can handle most of them, but before proceeding with the review of the work we would like to anticipate how we intend to set up the review so as to understand whether a manuscript revised in this way might be suitable for possible publication.
One of the critical issues raised by both the Reviewers concerns the validation of the prediction tool. As suggested by the Reviewers this would require comparing the outcomes of the proposed approach with the outcomes of the reordering policy implemented in the department over a long period. Unfortunately, this is not possible for the following reasons: (i) during the project we had access to a partial set of information only (heterogeneous data from different applications, data on therapies in paper format only, data relevant to the orders available for a limited period and for a subset of drugs only); (ii) the department over time underwent significant changes (it was relocated from one facility to another, expanded in terms of the number of beds, and changed the type of surgical patients treated); (iii) currently, since the project is over, we no longer have access to the data.
Because of the critical issues mentioned in points (i) and (ii), it was not possible to automatically extract and process the data within the timeframe of the project. Hence, in agreement with the department management, it was planned to (1) manually integrating and cleaning data coming from heterogeneous data sets (most of which were in paper format) to create a data set from which to derive empirical probabilities; (2) use these empirical probabilities to feed a drug demand generator.
Thus, the drug demand generator is an original contribution of the project and we now realize we need to describe it more accurately in the revised manuscript. The data set built in the initial phase of the project was appropriately divided into a training set and a test set, and was used to validate the ability of the prediction tool to estimate: (i) the presence of microorganisms in patients (ii) the length of stay, two key elements to estimate the demand for drugs. Machine learning tools have been used in this phase, and all this phase of the work has been documented in the master thesis (written in Italian) of one of the authors of the manuscript. What we could now do in the revised work is, therefore, to give more space to the description of the generator by including: (i) the description of the process that led to the definition of empirical probabilities; (ii) the analysis that led to the stratification of patients; (ii) the use of machine learning techniques to predict the presence of microorganisms and the duration of hospitalization. With the data currently available to us, no further analysis is possible.
The review of the work also will not be able to address Reviewer 1's critical issue related to the use of robust optimization. We believe that the implementation of robust solutions is a stand-alone contribution, to be addressed after a thorough study of the nominal problem that is the subject of this work.
Finally, we would like to clarify that in our approach, although the point of view of various stakeholders involved is considered, the decision maker is unique, and therefore it is not possible to apply the methods of game theory and bilevel programming suggested by Reviewer 2 which are used in the presence of multiple decision makers.
We apologize for this long letter but before proceeding to a review of the work we wanted to clarify what we can manage to avoid wasting your time with an inadequate review.
We conclude by saying that due to the amount of work required and the concomitance of the holiday period, we will not be able to complete the revision by August 15th, and for this reason we kindly ask for an extension of 6 weeks.
Sincerely, XXXXXX

Here is the answer we got from the Academic Editor:

Dear XXXX,
I trust all is well with you.
Please be informed that I have received a response from the Academic Editor regarding your concern. Kindly see below email for your reference.
=======================
About the email of authors, I would like to emphasize a few points:
Firstly, I found the explanations provided by the authors comprehensive and somehow persuasive. They need to mention them in their reply to the reviewer's comments and the revised manuscript. Especially about the accuracy of the prediction tool, authors need to reflect in the paper and possibly provide an expert validation – discussing the results with the experts or the problem owner (e.g., the empirical probabilities). In the revised manuscript, please also clearly explain the data extraction (and data sources) and how that is used in the model. Please also add a discussion addressing the (relevance) of robust optimization and game-theoretical studies and how they may add value (and could be considered future research directions).
It is also acceptable if authors need some more time to address the comments and revise the manuscript.
========================
Thank you and if I could be of any help, please let me know.

Finally, hereafter we provide a detailed answer to all of your concerns.

Table 1 responses to the reviewers' comments

	**Reviewer 1 - Comments to the authors**	**Authors' responses**
**R1.1**	The paper would be greatly improved if it included a direct quantitative and qualitative comparison of the proposed solution with what has been currently implemented in practice. My biggest concern relates to the accuracy of the forecasting tool and thus I strongly suggest a one-to-one evaluation of the inventory planning framework with the results obtained at the hospital in the same period. In other words, the authors could run a hypothetical retrospective "trial" in which they use for some period of time the proposed system in parallel to what is currently implemented in the clinic. This analysis would highlight the true benefit and applicability of the decision support tool.	*We thank the reviewer for this critical comment.*<br>*We acknowledge that such a comparison would significantly strengthen our contribution but, unfortunately, we cannot compare our results with those of the Department under study for several reasons: (i) during the project, we had access to a partial set of information only (heterogeneous data from different applications, data on therapies in paper format only, data relevant to the orders available for a limited period and for a subset of drugs only); (ii) the department underwent significant changes (it was relocated from one facility to another, expanded in terms of the number of beds, and changed the type of surgical patients treated); (iii) currently, since the project is over, we no longer have access to the data.*<br><br>*Nonetheless, in (new) Section 6.2, we have compared orders issued by the ward in June 2016, with the orders associated with the optimization model solution for the same period. Such a comparison shows that the implementation of the model would provide significant benefits.*
**R1.2**	A suggestion would be to add a section regarding the deployment and implementation of the tool in practice. What are the challenges that need to be overcome for its integration?	*We thank the reviewer for this suggestion. We describe the tool we developed in (new) Section 7. Such a tool (i) allows a rapid*

<table>
<tr><td></td><td>How would the interface for the decision maker look like? This addition in combination with the one-to-one comparison will significantly improve the manuscript.</td><td>transcription of the patient therapies; (ii) helps clinicians and nursing staff in data entry activities. The application uses the Swing Java library and follows a top-down event-driven approach: the main graphical area contains general information on a certain patient and is organized in panels containing specific information that are filled when some event occurs. Additional functionalities could be embedded in the tool but their implementation has still to be done.</td></tr>
<tr><td>R1.3</td><td>I would recommend to modify the abstract and emphasizing more the key findings and conclusions of the investigation. The current structure is not very coherent.</td><td>The abstract has been updated, we hope that in the present form it looks more informative and coherent.</td></tr>
<tr><td>R1.4</td><td>Why does the forecasting tool not take into consideration the patients that are scheduled to be admitted to the hospital in the planning period in addition to those currently hospitalized? Scheduled surgeries for example depending on their severity are associated with the risk of ICU admission. Thus, they could be factored as part of the forecasting tool.</td><td>We apologize for not having pointed it out in a clear way, but the forecasting tool actually considers those patients. We hope we have made it clear in this revised version of the manuscript.</td></tr>
<tr><td>R1.5</td><td>The section regarding the demand generation process and forecast needs to be substantially refined and expanded. The authors should discuss how they learn the probabilities of the proposed flowchart and in which ways the patient’s history and current medical conditions are used to adjust the estimation. Figure 1 shows a very simplified and abstract version of the generator. Technical details are missing and thus the analysis cannot be neither evaluated nor replicated.</td><td rowspan="3">We thank the reviewer for these suggestions. We omitted several details in our first submission and we have now thoroughly rewritten the paper in a way that is richer in details and informative (see e.g. new Section 4).
Specifically, in (new) Section 4.1, we have carefully explained how data were collected indicating the (5) different data sources we accessed, the time-frame these data refers to, and the stakeholder involved in the data collection process.
In (new) Section 4.2 we thoroughly describe how data were analyzed to stratify patients (Section 4.2.1.), to predict their length of stay (LoS) (Section 4.2.2) and predict infection outbreaks (Section 4.2.3). The LoS and infection outbreak predictions were carried out using machine learning techniques as suggested by the reviewer. The LoS prediction accuracy was assessed using MAE.
In Section 4.2.4 we describe how we calculated the empirical distributions subsequently used in the demand generator.
Finally, in (new) Section 4.3, we thoroughly describe the demand generator. In this section, we have improved Figure 1 as requested by the reviewer and added Figure 2. Specifically, Figure 1 focuses on the ward, whereas Figure 2 focuses on patients. We hope that these new figures allow a better understanding of the demand generator.</td></tr>
<tr><td>R1.6</td><td>Given the data that is available to the authors, I would recommend the use of machine learning techniques to estimate the future trajectory and the demand of medication at the personalized level. These methodologies do not require probabilistic assumptions and are able to devise complex, non-linear rules that result in accurate demand estimations.</td></tr>
<tr><td>R1.7</td><td>How are the authors evaluating the accuracy of the forecasting methodology they propose? A retrospective analysis of this approach using data from the healthcare system in Italy would provide significant evidence regarding the validity of the proposed generator.</td></tr>
<tr><td>R1.8</td><td>To account for the uncertainty in the demand estimation, I suggest to the authors to consider uncertainty sets and optimize against the worst-case scenario. By using robust optimization, they could yield better solutions that protect the hospital system against forecasting errors. For a review of robust optimization approaches, they can resort to the</td><td>We thank the reviewer for this suggestion. However, based on our previous experience in robust optimization, we believe that the implementation of robust solutions is a stand-alone contribution, to be addressed after a thorough study of the nominal problem. In this</td></tr>
</table>

	work of Bertsimas et al.: Bertsimas, D., Brown, D.B. and Caramanis, C., 2011. Theory and applications of robust optimization. SIAM review, 53(3), pp.464-501.	*paper, we address the nominal problem whereas the robust version of the model will be certainly the object of our future research.*<br><br>*In agreement with the Academic Editor we decided not to address this comment.*<br><br>*Nonetheless, we added in (new) Section 4.4 some hints on alternative approaches to uncertainty.*
**R1.9**	The authors should provide a Github repository where researchers can review and access the code and the data from the paper.	*We thank the reviewer for this suggestion, we have now created a Github repository where we have shared the code used (https://github.com/pcfvmn/PLOSONE). Please, note that to run the simulation and optimization models, a full version of Rockwell Arena 14.0 is needed. To solve the optimization model embedded in the simulator CPLEX 12.6 is needed as well. In addition, it is worth pointing out that such a simulation environment was designed as a test bench to be used for research purposes not as a distributable user-friendly software.*
**R1.10**	The introduction is barely citing other papers that support the authors' claims. My suggestion would be to add references to other investigations that highlight some of the information that is provided by the authors regarding the drug replenishment at the ICU setting.	*References were added that advocate the use of tools predicting the evolution of patient's critical conditions [23][19][42].*
**R1.11**	The first paragraph of the introduction can be split into two paragraphs that capture the two different main ideas discussed in this section.	*Thank for your comment. We have now split the paragraph to separate problem description from motivations.*
**R1.12**	*It would be very helpful to the reader to provide an Algorithm overview or a visualization of the entire approach as described in the Introduction.*	*We thank the reviewer for this suggestion. A visualization of our approach can be found in Fig.3 and Fig.4 in Section 5. We refer to these figures in the introduction of (new) Section 5.*
**R1.13**	The authors could consider breaking some of the longer sentences in two to help the reader understand better the content of the paper (see Examples in the Introduction and Conclusions).	*Fixed, thank you.*
**R1.14**	Line 6: Typo – makes it possible continuous review policies.	*Fixed, thank you.*
	**Reviewer 2 - Comments to the authors**	*Authors' responses*
**R2.1**	The paper is missing a proper validation section. This is important.	*We acknowledge that such a comparison would significantly strengthen our contribution but, unfortunately, we cannot compare our results with those of the Department under study for several reasons: (i) during the project we had access to a partial set of information only (heterogeneous data from different applications, data on therapies in paper format only, data relevant to the orders available for a limited period and a subset of drugs only); (ii) the department over time underwent significant changes (it was relocated from one facility to another, expanded in terms of the*

		number of beds, and changed the type of surgical patients treated); (iii) currently, since the project is over, we no longer have access to the data.<br><br>*Nonetheless, in (new) Section 6.2, we have compared orders issued by the ward in June 2016, with the orders associated with the optimization model solution for the same period. Such a comparison shows that the implementation of the model would provide significant benefits.*
**R2.2**	Fig. 1 is not clear, e.g., the path from “Clinician expertise” to “Other Lab Issue” is a Possible death! …<br>So, I suggest that the flow chart have two exit nodes. One exit is due to death and the other exit is due to (proper) treatment.	*We thank the reviewer for this critical comment.*<br>*Former Figure 1 has been replaced by new Figure 1 and Figure 2. Figure 1 focuses on the ward, whereas Figure 2 focuses on patients. We hope that these new figures allow a better understanding of the demand generator.*
**R2.3**	Hierarchical optimization is a classical problem in Operations Research. E.g., this is an old classical reference:<br>Anandalingam, G., & Friesz, T. L. (1992). Hierarchical optimization: An introduction. Annals of Operations Research, 34(1), 1-11.<br>So, in general, there is no adequate literature review (in the paper) about hierarchical optimization. The aim of this literature review should be to identify the state-of-the-art hierarchical optimization methods to implement one of them (in the paper). Note the implementation of hierarchical optimization in the MILP model is not the state of the art. This is a weak point in the paper.	*We thank the reviewer for this critical comment, and we apologize if the paper was written in a way that could be misunderstood. However, we would like to clarify that in our approach, although we consider the point of view of different stakeholders, we assume that the decision-maker is unique, and therefore it is not possible to apply the methods of game theory and bilevel programming you suggested as they are used in the presence of multiple decision-makers/obectives.*<br>*In agreement with the Academic Editor we decided not to address this comment.*
**R2.4**	In section 5.3, the authors state the following:<br>"The number of entities to create is determined by sampling from suitable empirical distributions based on the data collected on-site during the observation period."I do not understand the term: "suitable empirical distributions".<br>Moreover, I think it is better first to fit the empirical distribution to a "suitable" theoretical distribution; then to use this theoretical distribution in the simulation model.	*We thank the reviewer for this critical comment. We used empirical distribution as it carries two main advantages (Law and Kelton, 2000, page 296): (i) it allows avoiding the occurrence of fairly large (or small) values that might not practically occur in reality; (ii) it allows for better capturing the characteristics of the data when theoretical distributions display poor fit. Unfortunately, no theoretical distributions displayed a decent fit with our data.*
**R2.5**	Also in section 5.3, the authors state the following: "The algorithm is executed in shell (i.e., while the model runs, the simulation clock does not advance)," think the authors mean: (i.e., while the algorithm -- not the model -- runs, ...).	*Fixed, thank you.*
