# Supplementary material for: Rush order containment of critical drugs in ICUs
Source: PLoS One. 2022 Jun 23;17(6):e0264928. doi: 10.1371/journal.pone.0264928 (PMC9223342; doi:10.1371/journal.pone.0264928)
Supplement: Attachment — Submitted filename: reviewReportRev2_PLOSONE.pdf [file pone.0264928.s002.pdf]

Dear Editor,

Thank you for allowing us to review our work.
The table below shows a detailed response to the comments of the Reviewer, whom we warmly thank.
It is worth pointing out that due to the data access issues, of which we have informed the Academic Editor since the first review, we could only partially address comments R1.2 and R1.4.
We hope that the paper, in the present form, is now suitable to be published on PLOSONE.

Table 1 responses to the Reviewers' comments

	**Reviewer 1 - Comments to the authors**	**Authors' responses**
**R1.0**	Reviewer #1: I would like to thank the authors for their time and effort in conducting a major revision of the manuscript. The paper has been significantly improved as it now provides an indirect comparison of the model's proposed solution with the actual decisions implemented in practice in June 2016. Section 7 also illustrates an online version of the tool and discusses the implications and challenges associated with it in a real-world setting. The section regarding the demand generation process and forecasting has been expanded and the methodology proposed is now clearer to the reader. Below you will find my comments to the authors. I believe that with a minor revision the manuscript would be ready for publication.	*We warmly thank the Reviewer for her/his encouraging words and, most of all, for having helped us to significantly improve our manuscript.*
**R1.1**	In Section 4.1 the authors outline in detail the data sources that they used for their study. Note that Prosafe data were recorded between 01/2011-04/2016 and miss information during the stay. Microbiology data refer to a 6 year period from 01/2010-02/2016 and not a 5 year period as indicated in the paper. The authors also used 69 digitized admissions from June 2019. How was this information subsequently consolidated? Based on the information currently available in the paper, the authors could use the information for patients present in both the Prosafe data and the Microbiology data. However, the rest could not be combined. In addition, the 69 digitized admissions do not refer to the same period (2010-2016) and thus no information from Prosafe and microbiology could be applied for those patients. What was the resulting size of the dataset? How did the authors deal with missing values? It would be great to provide a Table summarizing the mean, standard deviation, and missing percentage for the final dataset. Perhaps this information could go into the appendix, but it remains critically important for the reproducibility of the study.	*The Reviewer is right and we apologize for not clearly having explained the consistency of the databases in the previous version of the manuscript. We have added an explanation at the end of Section 4.1 which we quote here for completeness. "The 5 data sources feed two datasets. The first, consisting of 2502 admissions, is the result of combining, in the same time horizon, data from Prosafe and the microbiology laboratory. This dataset is used in Section 4.2 for data analysis and specifically, for patient stratification and prediction of Los and infection outbreak. The second dataset instead, is composed of 66 patients, corresponding to 69 admissions, and is obtained by cross-referencing manually collected data on therapies and drug orders. This dataset is used to recover the drug demand of a specific month and to obtain a first comparison between the optimized solution and the real case. The related results are shown in Section 6.2."*

**R1.2**	I would suggest the authors to present the R^2 of their computational experiments in predicting length of stay as it provides a comparison with a simple baseline. Perhaps they could consider the average length of stay for each class as the baseline and then analyse the improvement they achieve using the SVR kernel regression.	*We agree with the Reviewer that presenting R2 would give immediate insight into the goodness of the model. However, for the reasons we have explained above, unfortunately, the version of the database we currently have is not exactly aligned with the one on which the results presented were obtained. In fact, the database underwent modifications following interaction with physicians who might suggest that some patients should be considered outliers or that some features should not be considered. Thus, we cannot re-run the scripts to calculate the new metric. However, we see that in the literature the use of Mean Absolute Error (MAE) to assess the goodness of SVR is quite widespread (as for example done in the health care setting in Zhang, B., Ren, H., Huang, G. et al. Predicting blood pressure from physiological index data using the SVR algorithm. BMC Bioinformatics 20, 109 (2019)). In addition, in the revised manuscript, Table 5 has been reorganized and we added a column giving the LoS range returned by classification. In defining classes, after extensive discussion with physicians, outliers were excluded from the dataset.*
**R1.3**	Please add a citation for the Relieff features ranking methodology.	*Done. The citation has been added*
**R1.4**	The mean of sensitivity and specificity might be misleading for the interpretation of the results. The authors can report instead in Table 6 in different columns the average sensitivity, specificity, and accuracy for a given threshold as well as the overall out-of-sample AUC. The currently presented results are not very insightful from a statistical perspective. In addition, confidence intervals are needed around this metrics.	*We agree with the Reviewer that in a binary classification problem, accuracy is given by the mean of sensitivity and specificity only if the two classes (patients with infection – "yes" label - and patients without infection – "no" label) are equally represented. While it is not true that among hospitalized patients 50% are infected, it is true that in the available dataset of patients for whom physicians suspect infection and request laboratory tests, the percentage of infected patients was just about half. Specifically, the dataset used for this experimentation relates to data before 2014 as explained in the text and consists of 296 patients and 213 features; among them, 154 were infected patients and 142 were not infected. For this reason, at that time, it was chosen to adopt the mean of sensitivity and specificity as a metric to define the accuracy of the SVM model. We fully agree that the results present in the work on predicting the infection outbreak are very preliminary and much work should be done in that direction. However, the emphasis of this work is not on prediction. In addition, when talking with physicians, it emerged that it would be much more useful for them to be able to predict the type of infection (hence regression) rather than the infection outbreak (hence classification). However, there was little data available to perform such an analysis.*

		It would be extremely interesting to deepen this line of research with different databases, following the Reviewer's suggestions.
**R1.5**	I think it would greatly improve the paper if the authors could add a graphical illustration of the steps necessary to apply the tool. In other words, the timing and sequence of using the forecasting tool and the simulation in combination with the optimization. Parts of that are already described in Section 4.3. This would be a more high-level implementation and workflow of the system compared to Figure 3.	*The illustration has been added, see Figure 3. Note that, when applying the tool, only prediction and optimization are required. In particular, the new figure shows the action flow on the first day of each period, when prediction, based on current patient data, is computed and used to feed the optimization module, yielding the push order agenda. On any other day of the period, scheduled orders are issued and fulfilled with a certain lead time, while stockouts trigger rush orders that are fulfilled on the same day.*
**R1.6**	What are the implications in terms of cost other than just frequency from the comparisons conducted in Section 6.2? By having better planned and less rush orders what would be the approximate impact on the costing of the department?	*We thank the Reviewer for this interesting comment, which allowed us to briefly discuss the additional benefits of optimized drug management beyond the obvious ones resulting from rush order containment. Some comments on this are included at the end of Section 6.2 of the revised version of the manuscript.*
